# Artificial Intelligence in Brain Tumor Imaging: A Step toward Personalized Medicine

**DOI:** 10.3390/curroncol30030203

**Published:** 2023-02-22

**Authors:** Maurizio Cè, Giovanni Irmici, Chiara Foschini, Giulia Maria Danesini, Lydia Viviana Falsitta, Maria Lina Serio, Andrea Fontana, Carlo Martinenghi, Giancarlo Oliva, Michaela Cellina

**Affiliations:** 1 Postgraduation School in Radiodiagnostics, Università degli Studi di Milano, Via Festa del Perdono 7, 20122 Milan, Italy; 2 Postgraduation School in Radiodiagnostics, University of Rome Tor Vergata, Viale Oxford 81, 00133 Rome, Italy; 3 Radiology Department, San Raffaele Hospital, Via Olgettina 60, 20132 Milan, Italy; 4 Radiology Department, Fatebenefratelli Hospital, ASST Fatebenefratelli Sacco, Piazza Principessa Clotilde 3, 20121 Milan, Italy

**Keywords:** artificial intelligence, brain tumors, glioblastoma, deep learning, prognosis prediction

## Abstract

The application of artificial intelligence (AI) is accelerating the paradigm shift towards patient-tailored brain tumor management, achieving optimal onco-functional balance for each individual. AI-based models can positively impact different stages of the diagnostic and therapeutic process. Although the histological investigation will remain difficult to replace, in the near future the radiomic approach will allow a complementary, repeatable and non-invasive characterization of the lesion, assisting oncologists and neurosurgeons in selecting the best therapeutic option and the correct molecular target in chemotherapy. AI-driven tools are already playing an important role in surgical planning, delimiting the extent of the lesion (segmentation) and its relationships with the brain structures, thus allowing precision brain surgery as radical as reasonably acceptable to preserve the quality of life. Finally, AI-assisted models allow the prediction of complications, recurrences and therapeutic response, suggesting the most appropriate follow-up. Looking to the future, AI-powered models promise to integrate biochemical and clinical data to stratify risk and direct patients to personalized screening protocols.

## 1. Introduction

Even though artificial intelligence (AI) is far from being used routinely in the current workflow of radiologists, the number of clinical studies using radiomics and radiogenomics approaches in neuroradiology is increasing day by day. In this article, we describe some examples of AI applications in the main activities related to brain tumor imaging, with a special focus on gliomas. These applications include lesion detection, differential diagnosis, non-invasive molecular characterization, the definition of lesion boundaries and spatial relationships (segmentation), and an assessment of response to therapy and prognosis. It is likely that in each of these areas, AI models will soon play a central role in assisting the radiologist in his daily work [1].

Gliomas are the most common type of central nervous system (CNS) neoplasm and arise from glial cells [2]. They represent a clinically and biologically heterogeneous disease, with several recognized histotypes and molecular subtypes, and a clinical history ranging from slow growth and predominantly benign prognosis, such as pilocytic astrocytoma, to particularly aggressive histological subtypes, such as glioblastoma multiforme (GBM), which is associated with rapid progression and poor prognosis [3,4]. Therefore, timely and accurate diagnosis is essential to ensure adequate patient treatment and longtime survival.

Historically, brain tumor classification has been solely based on histopathological features [5], whereas the latest editions incorporate genetic and epigenetic information, such as molecular markers (e.g., IDH mutation, 1p/19q codeletion, etc.) and DNA methylation profiles [6,7]. The genetic and epigenetic makeups define the molecular signature, a “barcode” of the tumor, whose recognition is essential for clinical decision-making in the era of targeted therapies [8]. Therefore, tissue sampling remains the gold standard for decoding the molecular landscape of most CNS tumors, especially for gliomas [9]. Nevertheless, growing evidence has highlighted the powerful role of artificial intelligence in oncological neuroimaging through the extraction of quantitative information from routine radiological examinations [10]. Alongside the molecular signature is the imaging signature, which offers complementary and ideally additional information for the characterization of the brain tumor, with a potential role in guiding the choice of the most appropriate therapy and clinical management [11]. In this landscape, AI-assisted tools represent the bridge from precision diagnostics to precision therapeutics [12].

**Figure 1:**
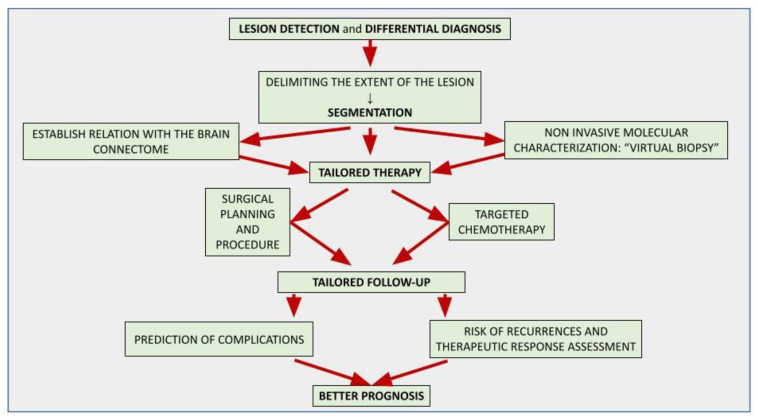
The flowchart in Figure 1 represents the developed AI tools for brain tumor imaging and their aim. The final purpose is to provide customized therapy and follow-up for each patient in order to achieve a good outcome.

In Figure 1, the flowchart shows the possible applications of AI in brain tumor imaging to provide customized patient management.

## 2. An Introduction to Artificial Intelligence and Related Concepts

In this section, we provide some basic definitions and theoretical frameworks of the most important AI-related concepts in biomedical imaging.

### 2.1. Artificial Intelligence (AI)

AI can be defined as technology that mimics human cognitive processes, such as learning, reasoning, and problem-solving. Developed as a branch of computer science, present-day AI is a broad field of knowledge that welcomes contributions from different disciplines, such as statistics, informatics, and physics.

### 2.2. Radiomics

Radiomics was first described by Lambin in 2012 as the high-throughput extraction of numerous quantitative image features from radiographic images for diagnostic purposes [13]. At the basis of this new approach is the awareness that radiological images must be considered as numerical data rather than simple images, providing much more information than can be perceived by the radiologist through a qualitative evaluation [14,15,16]. The radiomic paradigm seeks to extract quantitative and ideally repeatable information from diagnostic images, including complex patterns that are challenging for the human eye to detect or quantify. There are high expectations that the contribution of artificial intelligence to biomedical imaging will help close the gap towards personalized medicine [17,18].

#### 2.2.1. Radiogenomics

Radiogenomics may be considered a subset of radiomic applications, aiming to link imaging and biology, thus correlating lesion imaging phenotype (“radio“) to the genotype (“genomics”), based on the assumption that phenotype is the expression of genotype; thus, genomic and proteomics patterns can be expressed in terms of macroscopic image-based features [19].

#### 2.2.2. Radiomics Workflow

Radiomics workflow is a complex process leading to the development and validation of AI-based tools aiming to extract diagnostic and/or predictive information from biomedical images [20,21]. It includes some well-established steps for image acquisition (or data collection), post-processing/reconstruction, segmentation (definition of the area/volume of interest), feature extraction and harmonization, and data mining/model building. The last phase consists of the effective extraction of valuable information from imaging data, essentially through machine learning (ML) methods that need to be trained, validated, and tested to ensure reliability and clinical applicability [22,23].

#### 2.2.3. Machine Learning

ML is a branch of AI aimed at the automated detection of meaningful patterns in data and is at the basis of data mining [24,25,26,27]. In radiology, ML can be used to extract information from imaging data [28]. First, ML algorithms are trained to perform a certain task related to medical images starting from some initial data, which must be provided in a certain way, depending on whether the model is supervised or unsupervised. In the second phase, the computational power of modern computers is exploited to perform these tasks automatically or semi-automatically in order to replace or improve the performance of the human decision-maker. There are essentially four main types of ML (supervised learning, unsupervised learning, semi-supervised learning, and self-supervised learning). Supervised learning (SL) and unsupervised learning (UL) are the two most used in radiomics applications [29].

#### 2.2.4. Supervised Learning

Supervised learning (SL) is the most basic mechanism of ML and, as the name suggests, needs some degree of human supervision to be trained [25,26,27,30]. SL algorithms simulate the human cognitive process of “learning by examples”. This kind of ML is appropriate for very general classification tasks where new elements must be labeled in accordance with a set of predefined categories. These techniques require a training dataset consisting of input-output (x, y) tuples composed of input (x) and corresponding label values (y). Human experts annotate (“label”) input data points with corresponding label values (for example, classifying a brain mass as “tumor” or “nontumor”) to create the labeled data points in order to train the algorithm. While normally a computer is programmed to perform a known operation on the input to obtain the output, in this case the computer is asked to find the operation that links the given input and output. In this case, the algorithm is structured to test a series of theoretical hypotheses mapping possible relationships (x → y) among the data, mimicking the human annotator who learns through experience to infer the category of appearance based on image characteristics.

#### 2.2.5. Unsupervised Learning

Unsupervised learning (UL) algorithms rely solely on the inherent structure of data that have not been labeled, classified, or categorized by an expert and exhibit self-organization to capture hidden patterns in data [26,30]. In this type of ML, the learning algorithm is given a naive dataset and instructed to extract knowledge from it. They are utilized in tasks such as clustering, where the goal is to divide the dataset into groups based on particular feature characteristics, and association, where the goal is to identify rules that link data points together.

##### Semi-Supervised Learning

Semi-supervised learning refers to a machine learning paradigm intermediate between unsupervised and supervised learning that works primarily on unlabeled data with a small amount of labeled data [26,30].

##### Self-Supervised Learning

Self-supervised learning refers to a machine learning paradigm in which the basic idea is to automatically generate some kind of supervisory signal to solve some task [26,30]. Self-supervised learning is very similar to unsupervised since no labels are given, but it aims to tackle tasks that are traditionally done by supervised learning. Since it is not possible to provide adequate supervision for all the data a large project must accumulate, this type of ML addresses the problem of low data availability by taking advantage of the abundant amount of accessible but unlabeled data, in order to train precise classifiers. Some neural networks, for example, autoencoders, are sometimes called self-supervised learning tools.

#### 2.2.6. ML Models

At the heart of every ML approach are models that can extract insights from unseen datasets to find patterns or make decisions [26,30]. ML models are the protagonists of ML processes, i.e., they are those who learn in the training phase and who return this knowledge when put to the test in an unprecedented context. ML models can be defined as a set of rules for manipulating data according to theoretical hypotheses that map the possible relationships within the dataset. We generally distinguish models based on statistics and models based on artificial neural networks. Statistical assumptions have the characteristic of being computationally feasible, which means that they can be easily translated into programming code to perform automated data analysis. Through the automation of machines, it is possible to test the feasibility of a model to infer the relationship within a given dataset and compare the performance of different models. Of course, different assumptions or mapping models can be used to infer predictions of an amount of interest that satisfies a predefined requirement, which in the simplest case of ML is the relationship between the native image and the label that the radiologist assigned. The validation and test phases, which follow the training phase, are necessary to evaluate the effectiveness of the mapping prescribed by the model (if any) within new data.

#### 2.2.7. Features

While radiologists primarily evaluate qualitative features, such as increase or decrease in signal intensity, by comparison with a subjective reference standard, radiomics features are quantifiable image properties, or metrics, that can be easily calculated or measured by a machine. In general, a distinction is made between hand-crafted features (manually defined by an expert) and automatically extracted features (usually through deep learning algorithms [DL], see below). Some features, called first-level features, include basic characteristics such as shape, volume, and intensity signal metrics, for which it is easy to guess their meaning from a qualitative point of view. On the other hand, second-order features are obtained from the combination of first-level features or automatically extracted using DL networks and may appear clinically uninformative to the radiologist [31]. Yet, it is especially from these high-level features that the potential of AI-based tools derives, which can capture what the radiologist does not. The feature extraction phase is fundamental in the radiomics workflow because they represent the raw material on which the artificial intelligence models are trained and validated. Therefore, the choice of reproducible and robust features is essential in the radiomic workflow [32].

#### 2.2.8. Artificial Neural Network

Artificial neural networks (ANNs) are a particular learning paradigm inspired by the human brain’s biological network [33,34]. The ANNs consist of layers of nodes, each representing a computational unit that processes the input according to a specific function and transfers the output, through a series of interconnections to one or more successive nodes. Although ANN nodes differ markedly from biological cells in terms of complexity and amount of connections, the whole of the network exhibits emergent properties, as does the biological network. These properties are generated by the coordinated activity of many smaller units, each performing an elementary computational operation, substantially adding up the inputs—variously weighted—and transferring the information through the neuron when a certain threshold value is reached. The learning capabilities of ANNs are not based on testing statistical hypotheses to map possible relationships between data, but on the flexibility of the computational properties of the neural ensemble.

#### 2.2.9. Deep Learning

Deep learning is a domain of AI that leverages sophisticated ANNs, such as convolutional neural networks (CNNs), to identify complex patterns in data, which is critical for image analysis [35,36,37]. CNNs are a key DL technique for automated radiomics feature extraction as they are capable of automatically extracting deep images, features discriminating infinitesimal details and handling a large amount of data [38]. DL networks are composed of a huge number of intermediate layers representing increasing degrees of sophistication. These models are inspired by the organization of the visual cortex of the mammalian brain, where the hierarchically organized layers process increasingly complex intermediate visual features, such as lines, edges, and shapes until they return to the meaning of the entire visual object. It is not intuitively decipherable how the processing of the middle layers adds up to the result; this is also known as the “black box phenomenon” and contributes to the challenge of interpreting the results of AI tools [39].

DL models are designed to capture the relation between local features and the entire image context, thus resulting in higher performance in image-recognition tasks. Some of the very popular CNNs, such as AlexNet [40], VGG [41] and GoogLeNet [42], are currently being used in medical image-classification tasks. Various network architectures or stacks of linear and nonlinear functions, such as CNNs or auto-encoders, are used in DL-based radiomics to find the most important/critical characteristics of radiological images. In 2019, the simplest form of a CNN was proposed to classify brain images into three classes (glioma, meningioma, and pituitary), and a classification accuracy of 84.19% was reported [43].

## 3. Lesion Detection and Differential Diagnosis

AI-powered tools can aid neuroradiologists in lesion detection and differential diagnosis.

Since gliomas are often diagnosed when they are large and symptomatic, the detection of glioma-like lesions on MRI may seem relatively trivial to an experienced neuroradiologist. Conversely, the early diagnosis of small brain metastases (BM) in oncological patients during follow-up is challenging, because sensitivity on MRI is variable, and many details of MRI acquisition can impact the performance [44]. However, since stereotactic radiosurgery protocols and other therapeutic decisions are based on the number and location of even small metastases, early diagnosis is a real concern for neuroradiologists, given the high impact on the patient’s prognosis. For this reason, most of the computer-aided detection (CAD) tools available in the field of neuro-oncology focus primarily on the automated detection of brain metastases.

The proper tuning of CAD tools is essential to ensure diagnostic accuracy, lowering the risk of overdiagnosis, overtreatment, and unreasonable concern in patients [23]. Generally speaking, if the threshold sensibility is too low, the model can be affected by a high false-positive rate, for example, including vascular structures instead of small metastases; on the other hand, when the threshold is high, the model can fail to detect small (in particular, <3 mm) lesions [45].

Park et al. have recently demonstrated how DL-based models significantly increase the diagnostic accuracy in the detection of small lesions by exploiting the integration of large amounts of MRI data: in particular, a DL model that combines 3D Black Blood and 3D GRE MRI sequences outperformed a DL model using only 3D GRE sequences in the detection of brain metastases (*p* < 0.001), yielding a sensitivity of 93.1% versus 76.8% [46].

Solitary BM and GBM can exhibit quite similar MRI features, such as post-contrast ring enhancement, necrotic core, and large peritumoral edema presenting with high signal on T2-weighted and FLAIR images [47]. Differentiating these two entities is essential, considering they are the most common brain tumors in the adult population and have quite different treatments [47]. Thus, several researchers have focused on this topic, showing the advantages of multiparametric MRI [48,49] and, more recently, evaluating the performances of different AI-based classifiers compared to expert neuroradiologists.

For example, Swinburne et al. investigated whether an ML algorithm including advanced MRI (advMRI) data from 26 patients can reliably differentiate between GBMs (n = 9), BM (n = 9), and primary central nervous system lymphoma (PCNSL) (n = 8). Their multilayer perceptron model performed well in discriminating between the three pathological classes. After adopting a leave-one-out cross-validation strategy, the model achieved a maximum accuracy of 69.2%, intermediate to that of two human readers (65.4% and 80.8%). However, the use of the same model for cases where human reviewers disagreed on the diagnosis yielded an increase of 19.2% incorrect diagnoses. No evaluation with an independent test cohort was carried out in this study, and this represents the main limitation of this study [50].

Since the contrast enhancement and local infiltration of white matter bundles are key features of high grade-gliomas (HGGs) [51], most ML and DL algorithms exploit radiomic features extracted on post-contrast T1-weighted 3D images or diffusion-weighted images (DWI) and related techniques, such as diffusion tensor imaging (DTI).

For example, a recent study based on DTI metrics, especially fractional anisotropy (FA) and ADC values, demonstrated that peritumoral alteration is different in these two entities, with GBM showing greater heterogeneity due to the infiltrative nature and aggressive tumor [1,52]

The combination of radiomic and non-radiomic features (clinical and qualitative imaging) has in some cases been shown to be better than using radiomic features alone. For example, a study by Han et al., established the importance of adding clinically relevant data (e.g., age and sex) and routine radiological indices (tumor size, edema ratio, and location) to build an AI-driven model to differentiate between GBM and BM from lungs and other sites using a logistic regression model; the integrated model was superior to the single model [53].

BM can be the first manifestation of a still unknown extracerebral malignancy; therefore, ML tools have been applied in the clinical scenario in which patients are found with brain metastases without a known primary site of cancer [54]. Metastases coming from different primary cancers show differences in the local environments and consequently exhibit different radiomic features [12]. Ortiz-Ramón et al. provided good results in differentiating metastases from lung cancers, melanoma, and breast cancers when they implemented an AI-driven model with two- and three-dimensional texture analyses of T1-weighted post-contrast sequences within a nested cross-validation structure after quantizing the images with multiple numbers of gray-levels to evaluate the influence of quantization [55].

Another challenging differential diagnosis is between GBM and PCNSL since these entities may show similar appearances on conventional MRI, especially when GBMs do not present a necrotic core and the enhancement is not confined to the peripheral area but is more homogeneous [56,57]. Generally, PCNSLs are treated with whole-brain chemotherapy and radiotherapy, while GBM commonly undergoes surgical resection before chemo-radiotherapy [58]; therefore, a proper diagnosis is mandatory.

A recent study by Stadlbauer et al. [59] analyzed the effectiveness of a multiclass ML algorithm that integrates several radiomic features extracted from advanced MRI (including axial diffusion-weighted imaging sequences and a gradient echo dynamic susceptibility contrast (GE-DSC) perfusion) and physiological MRI (protocol including the vascular architecture mapping (VAM) and the quantitative blood-oxygenation-level-dependent (qBOLD)) to classify the most common brain enhancing-tumors: (GBM, anaplastic glioma, meningioma, PCNSL, or brain metastasis). When compared to the human reader, the AI-driven algorithms achieved a better performance, resulting in superior accuracy (0.875 vs. 0.850), precision (0.862 vs. 0.798), F-score (0.774 vs. 0.740), and AUROC (0.886 vs. 0.813); however, the radiologists demonstrated higher sensitivity (0.767 vs. 0.750) and specificity (0.925 vs. 0.902).

The DL paradigm has evolved in recent years as a big data grinding machine and has replaced many conventional algorithms in the field of image analysis as well. Furthermore, the development of open-source web platforms for programming DL models has expanded the frontiers of collaborative research in the development and validation of new DL-based tools. A good example is provided by Ucuzal et al., who developed web-based DL software aimed at the differential diagnosis of brain tumors using the popular Python programming language and the dedicated Keras library. Their software accepts multiple formats of the images, such as .jpeg, .jpg, and .png, and can be used to classify the input MRI image datasets into three diagnostic classes: meningioma, glioma, and pituitary tumors [60].

CNNs have a significant drawback in that they underutilize spatial relationships between the tumor and its surroundings, which is especially detrimental for classifying tumors. K. Adu and Y. Yu recently proposed a dilated capsule network model (CapsNet model), which is an extension of the traditional CNN, to address this issue [61]. In this model, the “routing by agreement” layer in the dilated CapsNet architecture takes the place of the pooling layer in the current CNN architecture [61]. Afshar et al. proposed a modified CapsNet architecture for classifying brain tumors that incorporates additional inputs into its pipeline from tissues surrounding the tumor, without detracting from the primary target, yielding satisfactory results [62].

Most AI-based classification algorithms target supratentorial tumors. In the posterior fossa, on the other hand, the two most common lesions in the adult population are hemangioblastoma, a benign tumor of vascular origin with a good survival rate, and brain metastases [63,64]. Obviously, discrimination between these entities is crucial for patient management as once again the therapeutic approach is different. In this field the role of AI-based is not yet well defined, however, a recent study attempted the differential diagnosis of intra-axial lesions of the posterior fossa using different radiomic algorithms (CNN, SVM, etc.), with promising results [1,65].

In some cases, even the differentiation between tumoral and non-tumoral processes is not simple. Tumefactive multiple sclerosis lesions, infection, inflammation disease (paraneoplastic syndrome and autoimmune disease), cortical dysplasia, and even stroke may be confused with tumoral processes, and accurate differential diagnosis based only on the radiological appearance is impossible due to the overlapping radiological features [66].

For example, tumefactive multiple sclerosis is a great mimicker of HGG on conventional MRI. The use of an AI-assisted tool can help the neuroradiologist to improve the differential diagnosis [1]: a recent study by Verma et al. achieved good results in differentiating GBMs from PCNSL from tumefactive multiple sclerosis lesions using an in-house software called dynamic texture parameter analysis (DTPA), which incorporates the analysis of quantitative texture parameters extracted from dynamic susceptibility contrast-enhanced (DSCE) sequences [67]. A more recent study by Han et al. evaluated the performance of different radiomic signature models in differentiating between low-grade glioma (LGG) and inflammation using radiomic features extracted from T1-weighted (T1WI) and T2-weighted (T2WI) MRI images. The features were chosen after a *t*-test and statistical regression (LASSO algorithm) to develop three radiomic models based on T1WI, T2WI, and combination (T1WI + T2WI), using, respectively four, eight, and five radiomic features each. The T2WI and combination models achieved better diagnostic efficacy in both the primary cohort and the validation cohort, significantly outperforming radiologist assessments [68].

The main results of the above studies are listed in Table 1.

## 4. Tumor Characterization

In the era of molecular therapies, diagnostic neuroimaging should guide the diagnosis and treatment planning of brain tumors through a non-invasive characterization of the lesion, sometimes also called “virtual biopsy”, based on radiomic and radiogenomic approaches [11].

To date, most studies have challenged ML models to address very general classification tasks for brain tumors, such as differentiating between GBM and brain metastases [69,70]. However, more recently, researchers focused on the development of AI-driven tools, aiming to recognize the radiological signature of the tumor to provide a comprehensive analysis of the grading, genomic and epigenomic landscape of cerebral gliomas, which is extremely useful for decision-making towards a personalized medicine perspective. Therefore, several studies have been published in recent years where AI algorithms are challenged in increasingly specific classification tasks, such as differentiation within different subgroups of gliomas, for example, low-grade gliomas (LGGs) compared to high-grade gliomas (HGGs) [71,72]; isocitrate dehydrogenase (IDH) wild-type (IDH(−)) vs. IDH-mutated (IDH(−)) [73]; 1p/19q chromosomal arm deletion [74]; and others.

Several studies have focused on glioma grading. For example, Cho et al. used a radiomics approach to test the performance of various ML classifiers in determining the grading of 285 glioma cases (210 HGG, 75 LGG) obtained from the Brain Tumor Segmentation 2017 Challenge. The researchers extracted a large set of radiomic features from routine brain MRI sequences, including T1-weighted, T1-weighted contrast-enhanced, T2-weighted, and FLAIR. Three supervised ML classifiers showed an average AUC of 0.9400 for training cohorts and 0.9030 (logistic regression 0.9010, support vector machine 0.8866, and random forest 0.9213) for test cohorts [75].

In another study, Tian et al. investigated the role of radiomics in differentiating grade II gliomas from grade III and IV; they extracted radiomics features from conventional, diffusion, and perfusion arterial spin labeling (ASL) MRI. After multiparametric MRI preprocessing, high-throughput texture and histogram parameters features were derived from patients’ volumes of interest (VOIs). Then, the support vector machine (SVM) classifier showed good accuracy/AUC (96.8%/0.987) for classifying LGGs from HGGs, and 98.1%/0.992, respectively, for classifying grades III from IV. Furthermore, they proved that texture features were more effective for non-invasively grading gliomas than histogram parameters [76].

Mzoughi et al. proposed a fully automatic deep multi-scale 3D CNN architecture for MRI gliomas brain tumor classification into low-grade gliomas and high-grade gliomas, using the whole volumetric T1 contrast-enhancement MRI sequence. For effective training, they used a data augmentation technique. After data augmentation and proper validation, the proposed approach achieved 96.49% accuracy, confirming that adequate MRI pre-processing and data augmentation could lead to the development of an accurate classification model when exploiting CNN-based approaches [77].

Chang et al. used CNNs for the differential diagnosis between IDH-mutant and IDH wild-type gliomas on conventional MRI imaging, achieving 92% accuracy; these results were in line with prior hypotheses based on visual assessment and underlying pathophysiology, as IDH wild-type lesions are characterized by more infiltrative and ill-defined borders. Furthermore, the authors found that nodular and heterogeneous contrast enhancement and “mass-like FLAIR edema” could aid in the prediction of MGMT methylation status, with up to 83% accuracy [78].

In another study, Kim et al. aimed to evaluate the added value of radiomic features extracted from MRI DWI and perfusion sequences in the prediction of IDH mutation and tumor grading in LGGs. For the IDH mutation, the model trained with multiparametric features showed similar performance to the model based on conventional sequences, but in tumor grading, it showed higher performance. This trend was confirmed in the independent validation set, demonstrating that DWI features and especially the apparent diffusion coefficient (ADC) map play a significant role in tumor grading [73].

In one of the first studies in the field, Akkus et al. presented a non-invasive method to predict 1p/19q chromosomal arm deletion from post-contrast T1- and T2-weighted MR images using a multi-scale CNN. They found that increased enhancement, infiltrative margins, and left frontal lobe predilection are associated with 1p19q codeletion with up to 93% accuracy [74].

In a larger, recent retrospective study, Meng et al. specifically targeted ATRX status in 123 patients diagnosed with gliomas (World Health Organization grades II–IV) using radiomics analysis, showing that radiomic features derived from preoperative MRI facilitate the efficient prediction of ATRX status in gliomas, achieving an AUC for ATRX mutation (ATRX(−)) of 0.84 (95% CI: 0.63–0.91) on the validation set, with a sensitivity, specificity, and accuracy of 0.73, 0.86, and 0.79, respectively [79].

In another retrospective study by Ren et al., researchers focused on the non-invasive prediction of molecular status for both IDH1 mutation and ATRX expression loss in LGGs, exploiting a radiomic approach based on high-throughput multiparametric MRI radiomic features. An optimal features subset was selected using a support vector machine (SVM) algorithm and ROC curve analysis was employed to assess the efficiency for the identification of the IDH1(+) and ATRX (−) status. Using 28 optimal texture features extracted from multiple MRI sequences, the SVM predictive model achieved excellent performances in terms of accuracies/AUCs/sensitivity/specificity/PPV/NPV in the prediction of IDH1(+) (94.74%/0.931/100%/85.71%/92.31%/100%, respectively) and ATRX (−) within LGGs (91.67%/0.926/94.74%/88.24%/90.00%/93.75%) [80].

Recently, some more ambitious studies have investigated the diagnostic accuracy of a radiomic approach in evaluating both the grading and the complete molecular profile of cerebral gliomas [81]. For instance, Habould et al. integrated clinical and laboratory data into a completely automated segmentation-based radiomics tool for the prediction of molecular status (ATRX, IDH1/2, MGMT, and 1p19q co-deletion), also distinguishing low-grade from high-grade gliomas. The system provided an AUC (validation/test) of 0.981 ± 0.015/0.885 ± 0.02 for the grading task. The prediction of the ATRX (−) condition had the best results, with an AUC of 0.979 ± 0.028/0.923 ± 0.045, followed by the prediction of IDH1/2(+), with an AUC of 0.929 ± 0.042/0.861 ± 0.023, while they showed only moderate results for the prediction of 1p19q and MGMT status [82].

In a similar study, Shboul et al. performed a non-invasive analysis of 108 pre-operative LGGs using imaging features to predict the status of MGMT methylation, IDH mutations, 1p/19q co-deletion, ATRX mutation, and TERT mutations, achieving a good accuracy with AUC of 0.83  ±  0.04, 0.84  ±  0.03, 0.80  ±  0.04, 0.70  ±  0.09, and 0.82  ±  0.04 [83].

A recent study focused on the detailed analysis of the tumor landscape within HGGs, highlighting the outstanding potential of DL algorithms in the extraction of new imaging markers, otherwise impossible to evaluate visually or with traditional radiomics approaches. Calabrese et al. retrospectively analyzed preoperative MRI data from 400 patients with WHO grade 4 glioblastoma or astrocytoma, who underwent resection and genetic testing to assess the status of nine key biomarkers: hotspot mutations of IDH1 or TERT promoter, pathogenic mutations of TP53, PTEN, ATRX, or CDKN2A/B, MGMT promoter methylation, EGFR amplification, and combined aneuploidy of chromosomes 7 and 10. An AI-driven model was tested in the prediction of biomarker status from MRI data using radiomics features, DL-based CNN features, and a combination of both. The results showed that the combination of radiomics and CNN features from preoperative MRI yields improved non-invasive genetic biomarker prediction performance in patients with WHO grade 4 diffuse astrocytic gliomas [84].

The main results of these studies are listed in Table 2.

The performance of the succinctly presented prediction models indicates the potential to correlate computer imaging features with the types of molecular mutations in gliomas, demonstrating how the radiomics approach has the potential to complement histological assessment.

## 5. Segmentation

Tumor segmentation consists of image analysis and delimitation of the regions of interest (ROI) comprising the tumor, from a 2D or 3D acquisition [85].

Segmentation represents a critical process in different applications, including brain cancer detection and diagnosis, accurate and reliable quantification of the disease burden, with objective volumetric assessment, useful for follow-up.

Segmentation is also an essential step in the radiomics workflow since lesion delimitation is preliminary to the extraction of radiomics features [23].

Furthermore, an accurate definition of the tumor boundaries is essential for treatment planning of brain tumors, since both radiotherapy and surgical approaches must be strictly limited to the pathological tissue, preserving as much as possible surrounding critical structures (functional cortical epicenters, white matter bundles), to achieve the best onco-functional balance for each patient: while too aggressive resection can lead to a reduction in the patient’s quality of life, too cautious resection leads to an increased risk of recurrence after surgery or radiotherapy [86].

Therefore, after detecting a brain lesion and defining whether it is neoplastic or non-neoplastic, the pre-treatment work-up usually is completed by tumor segmentation. Although also CT can be used to detect and segment a brain lesion, MRI is the modality of choice thanks to its superior tissue contrast resolution and multiparametric nature. Both conventional MRI and advanced MRI play a role in this phase [87].

Segmentation can be manual, semi-automated, or fully automated. To make an accurate manual segmentation of a brain tumor, the neuroradiologist subjectively evaluates some qualitative features such as the solid, contrast-enhanced part of the tumor, the presence of necrotic foci, the non-contrast enhanced part tumor and perifocal edema [1]. However, this process is strictly affected by a high degree of inter-reader variability due to several limitations such as the challenge of solving the infiltration-edema relationship unambiguously, especially in lesions with poor contrast enhancement and infiltrative pattern. In this scenario, AI-assisted semi-automated and automated segmentation tools based on DL algorithms can reduce segmentation time and significantly increase reproducibility and efficiency, with consequently a better outcome for the patient.

ML-based brain tumor segmentation techniques are typically based on voxel-based features which are extracted from the volume of interest (VOIs) of the image [87]. Several segmentation approaches have been tested showing a wide range of performances [1,87]. Many ML algorithms have been developed and tested for automatic tumor segmentation; however, their efficacy however must be evaluated in a real-world scenario before being introduced in clinical practice [1]. (Many ML-based fast and trustworthy segmentation methods have been developed based on the differentiation of each image voxel, to determine whether it belongs to normal brain tissue, tumor lesion, or other pathological brain tissue changes such as edema. At present, the most reliable segmentation methods are based on DL, a subgroup of ML based on neural networks allowing more complex classification, particularly using convolutional neural networks. CNNs have a great performance with about 90% accuracy in voxel labeling [1].

The infiltrative growth pattern of certain gliomas represents a diagnostic challenge to both neuroradiologists and automatic segmentation tools. However, differentiating between neoplastic infiltration and perifocal edema is essential for pre-surgical or radiotherapy planning. This task is hardly achieved using conventional qualitative approaches but there are expectations that ML methods may help to better identify infiltrative tissue margins on preoperative MR images, thereby allowing for more targeted, extensive surgical resections, localized biopsies, and tailored treatment planning. Two recent studies respectively developed and refined a multivariate support vector machine approach, incorporating features from conventional and advanced MRI modalities to predict infiltrated peritumoral tissue with approximately 90% cross-validated accuracy [88,89]. Chang et al. developed a fully automated system to generate a non-invasive map of cell density useful for the identification of infiltrative margins of gliomas [90]. Considering that current surgical resection largely relies on the enhancing tumor alone, these promising methods may guide a more aggressive and extensive treatment.

The infiltration and extent of brain tumors can be estimated with features extraction from FLAIR and ADC maps with a voxel-wise logistic regression model, with a good prediction of potential future recurrence [1,12,91].

Several ML and DL-based segmentation methods have been developed and tested. In 2022, Akinyelu et al. published a survey in which they compare the most recently developed segmentation techniques based on ML, CNN, Capsule Networks (CapsNet), and Vision Transformers (ViT). Most of these methods are used for segmentation or classification tasks, which are strictly related since they both contribute to identifying the grade of a brain tumor and planning its best treatment [92].

At present, DL-based models have a greater impact on brain tumor segmentation and classification tasks compared to ML-based models. The most used DL-based technique is CNN in which the images represent a direct input into the network of data, generating translation-invariant and deformation-resistant features used for a more accurate segmentation process. CNN algorithms have negative sides such as the need for a large dataset for training and to correctly identifiable inputs of different rotations and transformations.

Most CNN networks can extract information only from 2D MRI images. However, some recent studies aimed to extract volumetric information in 3D MRI images using CNN models [77,92]. ViT-based models, for example, can be used for 2D and 3D image segmentation and classification. In some studies, they are combined with CNNs models to capture both local contextual features and global semantic features.

CapsNet-based tumor segmentation techniques have been proposed in the literature to address the downsides of CNNs methods. As previously mentioned CapsNets require smaller datasets to be trained in comparison with CNNs and consider the tumor surrounding tissues [92,93]. Even if most of the CapsNet-based techniques proposed in the literature are used for brain tumor classification, CapsNet-based models are also very useful for segmentation tasks, since they need small-scale datasets to train and require lesser computational complexity compared to CNN-based techniques.

Most brain tumor segmentation techniques found in the literature are based on pure ML-based or DL-based algorithms. Just a few studies used a hybrid technique, however with promising results [92,94].

Segmentation of brain tumors still remains a challenging task, especially when dealing with gliomas infiltrative growth pattern, future developments of AI systems may allow a more precise tumor definition and hopefully a progressive replacement of manual segmentation.

## 6. Prognosis

AI-assisted tools represent a novel frontier for the prediction of complications, recurrence, and therapeutic response in neuro-oncology, helping to outline the most appropriate follow-up and long-term treatment [12]. Looking ahead, AI-powered models promise to integrate clinical and laboratory data to stratify risk and build personalized screening protocols, such as what has been proposed for breast cancer [95].

Finding the clinical uses of ML algorithms in clinical practice and identifying the areas of clinical care that can be enhanced by artificially generated algorithms are thus the next steps in neuro-oncology imaging [96].

### 6.1. Prediction of Complications

It is well known that post-surgical complications depend on numerous variables, both fixed and dynamic, and some AI integration models have already been applied in fields other than neurosurgery [97,98,99]. For instance, Campillo-Gimenez et al. developed a ML program able to predict the occurrence of surgical site infection through the analyses of patient medical records [100]; similar algorithms were also used to predict complications such as venous thromboembolism and surgical site infection in patients undergoing anterior lumbar fusion, exhibiting an accuracy of 95%, significantly outperforming traditional statistical means [101]. Hopkins et al. predicted the development of infection in patients undergoing posterior spinal fusion, with a positive predictive value of 92.3% [102].

A recent review by Williams et al. [103] reported a few studies regarding the potential of AI integration in predicting the development of several typical post-operative complications in brain tumor patients, usually preventable, including venous thromboembolism [104], falls [105], hypoglycemia [74,106], adverse drug events [107], and pressure ulcers [108].

Prognostic value—Currently, poor overall prognostication of tumors is based on independent risk factors such as histological grade and clinical data; in addition, molecular subtypes play an important role in response to treatment and overall survival of brain tumors [12,109]: for instance, MGMT mutation in GBM can improve treatment response [110], and IDH mutation is an important prognostic factor for patients with improved survival rates compared to IDH wildtype glioblastoma [111,112]. Conventional survival prediction based on statistical models is valid at the population level but does not consider individual patient peculiarities and therefore may be inaccurate. Radiomic analysis provides a wide variety of additional imaging information which, together with clinical, biochemical, and histological data, can be used to develop more accurate predictive models in order to plan more personalized treatment and surveillance.

However, radiomics metrics are not currently widely adopted in current predictive models, despite their potential to capture underlying tumor biology and outcomes. Now only a few studies included artificial intelligence algorithms. One of these studies extracted about 60 radiomics features from traditional and advanced MRI metrics of glioma patients, including tumor volume, angiogenesis, peritumoral infiltration, and cell density, to predict the overall survival group (low, medium, and high) and molecular subtype; the predictors achieved an accuracy of about 80% and 76%, respectively, with the most predictive features being tumor volumes, angiogenesis, peritumoral infiltration, cell density, and distance to the ventricles [113]. Another study analyzed the performance of two-stage, multimodal, multi-channel 3D DL networks in predicting overall survival yielding an accuracy of up to 0.91 for high-grade glioma patients. The first stage used 3D technology to automatically extract imaging features from multimodal preoperative MRI, DTI, and resting-state functional MRI, while the second stage added the demographic and tumor-related features [1,114].

Recent results of several studies aimed to predict overall survival through AI-driven applications were reported in a thorough review published by Zhu et al. [115]. One of these studies, conducted by Sanghazni and coworkers [116], extracted texture, shape, and volumetric features from multimodal MRI data to validate an ML-based model for overall survival prediction in 173 patients with GBM performed for 2-class (short and long) and 3-class (short, medium, and long) survival groups, with a demonstrated prediction accuracy of 97.5% and 87.1%. The peritumoral environment, when combined across multiparametric sequences, may play a key role in predicting long-term vs. short-term survival for GBM patients, according to research by Prasanna and colleagues [117]. They looked at the role of radiomic features extracted from preoperative conventional MR images of the peritumoral brain zone in predicting long-term (>18 months) vs. short-term (7 months) survival in GBM patients.

In another study, Park and colleagues aimed to include diffusion- and perfusion-weighted MRI sequences together with conventional MRI and clinical data to develop an integrative AI–based model for prognostication of patients with newly diagnosed GBM; they showed that multiparametric MRI prognostic model including radiomic information and clinical predictors, exhibited good discrimination and performed better than the conventional MRI radiomics model or clinical predictors alone [118].

In another study, Grist et al. used various unsupervised and supervised ML models to determine new patient subgroups in relation to survival, based on MRI data, in particular perfusion, DWI, and ADC values. These models successfully determined two new subgroups of brain tumors with different survival characteristics (*p* <  0.01), which were subsequently classified with high accuracy (98%) by a neural network [119].

Tumor hypoxia is also known as a factor decreasing survival in GBM patients, and a study on GBM hypoxia-associated radiomics by Beig et al. [120] revealed that, when combining clinical features with radiomic features related to hypoxia, the concordance index for survival prediction rises in comparison to when using “generic” radiomic features alone.

Radiomic features can also be used to generate novel subgroups that may more closely align with the biology of gliomas [121,122]; although these studies are still preliminary, and conducted on relatively small sample sizes, they seem to be even more accurate than clinical models and molecular markers currently used in WHO classification. Prognosis can also be stratified by measuring the proliferative index of a tumor, such as Ki-67, linked to a worse outcome, and several studies are beginning to cover this aspect by using radiomic features [123].

### 6.2. Prediction of Recurrence and Follow-Up

Response Assessment for Neuro-Oncology, also known as RANO, criteria have recently been proposed for evaluating treatment response, which also includes clinical status and abnormalities in T2/FLAIR signal intensity and enhancing tissue [124]. However, the RANO criteria are still a limited tool for assessing treatment response, especially considering that they use two-dimensional subjective measurements and exclude advanced imaging modalities, such as MR perfusion. In light of this, Kickingereder et al. showed that an ANN model is more reliable than the current RANO-based assessment for determining the time to progression [125].

The risk of recurrence is dramatically linked to the radicality of the resection, which in turn depends on the correct evaluation of the margins of the lesion and on the ability to distinguish between perilesional edema and tumor infiltration: these aspects have already been discussed in the section precedent regarding segmentation.

Differentiating between tumor recurrence and post-treatment alterations is a difficult choice to make when planning glioma treatment. Radiation necrosis is frequently experienced three years after receiving the standard radiotherapy and chemotherapy combination regimen for glioma [126]. The ability of MRI qualitative analysis to distinguish between radiation necrosis and tumor recurrence is currently limited [127], and the use of artificial intelligence has not yet been able to fully characterize tumor heterogeneity [128,129].

Only a few studies have investigated this issue so far [130].

Together, available evidence proves that AI and especially DL-based volumetric assessment of tumor response is both feasible and clinically important in the prediction of a neuroimaging endpoint [45]. Regarding the alteration of the tumor immune microenvironment, immunotherapies in GBM also lack trustworthy radiological imaging evaluation techniques. In their groundbreaking study, Narang et al. extracted six imaging features that are connected to intra-GBM CD3 activity using T1-weighted post-contrast and T2-FLAIR images as well as T-cell surface marker CD3D/E/G mRNA expression data from GBM patients [131].

### 6.3. Tailored Therapeutics

Current standard treatment for glioblastoma consists of maximal safe resection followed by radiation and chemotherapy with temozolomide, whilst lower-grade gliomas may be treated with surgery and/or chemo-radiation. A few clinical trials are starting to assess the role of immunotherapy in the treatment of patients with glioblastoma, including some targeting specific molecular pathways such as EGFR [132]. Adjuvant therapy in the post-surgical phase may achieve maximal efficacy with the help of AI-driven evaluations. Although there are still no examples of AI-driven brain tumor chemotherapy protocols in routine clinical, some studies focused on the potential role of AI in optimizing the chemotherapeutic protocols at other primary tumor sites, achieving promising results [133]. Only recently, Yauney et al. described an ML program that could iteratively optimize chemotherapeutic dose in a simulated trial of GBM patients [134]. In the future, AI platforms may also predict response to immunotherapy, as well as optimize the dose and treatment protocol [135].

### 6.4. Progression vs. Pseudo-Progression

Pseudoprogression is defined as an increase in enhancement and/or T2/FLAIR signal abnormality on MRI within 12 weeks of radiotherapy or combined radiotherapy-chemotherapy, with spontaneous resolution or stabilization without change in management, occurring in 15–50% of patients with gliomas (MGMT-methylated and IDH-mutant tumors especially) [136]. Antiangiogenic medications, on the other hand, may cause pseudo-response, which is a sharp decline in enhancement brought on by altering the blood-brain barrier with little or no change in the progression of the infiltrating portion and overall survival [1]. Additionally, it has been demonstrated that new immunotherapy drugs trigger complex inflammatory reactions, which makes evaluating responses more challenging [132]. Differentiating between pseudo-progression and true progression is thus very difficult on MRI, and artificial intelligence is just beginning to solve this diagnostic dilemma, as several studies do successfully confirm [137,138]. On the other hand, a systematic review by Kim et al. [132] recently analyzed seven studies that suggest otherwise, maybe due to the inadequate size of training data, an inappropriate AI algorithm, or the substantial heterogeneity across the studies. Radiation necrosis is another effect that can take place any time after radiation therapy, usually, around 1–2 years after, and the key radiology tool in differentiating pseudo-progression or radiation necrosis from true progression is dynamic susceptibility contrast MR perfusion-weighted imaging [139]; however, PWI is unreliable in patients treated with immunotherapy [140]. Although many studies [115,137,141,142,143] succeeded in showing how AI models were able to use advanced MRI data in distinguishing pseudo-progression from true tumor progression, further research is needed to include AI-based models in everyday medical practice. Moreover, to date, there is no objective histological definition of pseudo-progression [144], indicating that even histology might not be the gold standard in differentiating pseudo-progression from true tumor progression.

The main results of these studies are listed in Table 3.

## 7. Limitations

This is not a systematic review but a narrative review, and therefore we included the articles that we thought to be most relevant, but we cannot exclude that other interesting articles on this topic have not been mentioned.

## 8. Conclusions

This review provides an overview of the AI applications in brain oncological imaging.

The development of CAD tools can increase diagnostic accuracy in the detection of small metastatic brain lesions, to enable early and correct treatment planning, especially stereotactic radiosurgery.

The AI-driven extraction of imaging features unavailable to the human eye is changing the approach to radiological image analysis and reporting, transforming it from a qualitative interpretation to an objective, quantifiable and reproducible task.

Segmentation is an essential step in planning surgery or radiation therapy, monitoring lesions, and even developing radiomics-based tools, as it is preliminary to the extraction of radiomic features. However, manual segmentation is extremely time-consuming, and therefore researchers worked with semi-automated or fully automated AI-based tools to help radiologists to assess in their daily practice, providing objective measurements of tumor burden as well as the characterization of its growth patterns.

The differential diagnosis of primary brain neoplasms can be challenging, particularly when dealing with PCNSL and HGG; in addition, tumefactive multiple sclerosis and other benign inflammatory and infectious disorders can mimic neoplastic conditions. Non-invasive techniques for accurate diagnosis based on artificial intelligence can revolutionize the approach to brain disorders, avoiding invasive biopsies and allowing the most appropriate treatment to start joyfully.

The so-called “virtual biopsy” is providing promising results, not only in differential diagnosis but also in the non-invasive characterization of tumor histotypes, to obtain increasingly personalized therapeutic plans.

The better is the characterization of a lesion, the better are the chances that clinicians have of identifying the most effective therapies and predicting complications, recurrences and progression.

All of these AI applications aim to achieve personalized medicine, improved patient outcomes, and increased survival.

The future development and progressive diffusion of these instruments will result in benefits for clinicians and patients, and in a personalized medical approach.

## Figures and Tables

**Table 1:** The table reports the main characteristics and findings of the studies focused on lesion detection and the differential diagnosis of brain tumors.

Author and Year	Country	N. Patients	Database	MRI Sequences and Clinical Data	AI Model	Task	Main Results	Main Limitations
Park et al. [46]	South Korea	188 (917 lesions)	Institutional brain MRI database	3D-GRE, 3D-BB	DL model based on 3D U-net	BM detection (3D-BB + 3D-GRE vs. 3D-GRE)	3D-BB + 3D-GRE model sensitivity = 93.1% 3D-GRE model sensitivity = 76.8%, (*p* < 0.001)	Single-center, retrospective study, small data size, 3D-BB sequences may have limited availability in MRI scanners, model mostly trained on patients with metastases
Swinburne et al. [50]	USA	26	Institutional brain MRI database	DWI, DSC, DCE	MLP (Multilayer Perceptorn) model using VpNET2	GBM vs. BM vs. PCNSL	Increase in 19.2% in correct diagnoses in cases where neuroradiologists disagreed	Manual tumor segmentation, sample size, no evaluation with an independent test cohort
Skogen et al. [52]	Norway	43	Institutional brain MRI database	DTI (FA and ADC)	Commercially available texture analysis research software (TexRAD)	GBM vs. BM	The heterogeneity of the peritumoral edema was significantly higher in GBMs (sensitivity 80% and specificity 90%)	Retrospective study, analysis of a single slice, the manual drawn of the ROI
Han et al. [53]	China	350	Institutional brain MRI database (two centers)	T1C, clinical data (age, sex), routine radiological indices (tumor size, edema ratio, location)	AI-driven model using logistic regression model	GBM vs. BM (lung cancer and other sites)	Combination models superior to clinical or radiological models (AUC: 0.764 for differentiation and 0.759 for differentiation between MET-lung and MET-other in internal validation cohorts)	Radiomic only based on T1-enhanced images, retrospective study, many small groups of metastases from other than lungs
Ortiz-Ramón et al. [55]	Spain	67	Institutional brain MRI database	IR-T1	RF model	Differentiate the primary site of origin of brain metastases	Images quantized with 32 gray-levels (AUC = 0.873 ± 0.064). differentiating lung cancer from breast cancer (AUC = 0.963 ± 0.054) and melanoma (AUC = 0.936 ± 0.070)	Small set of BM, single-center study,
Stadlbauer et al. [59]	Austria	167	Institutional brain MRI database	Standard MRI (FLAIR, T1C), advanced MRI (DWI, DSC), physiological MRI (VAM = vascular architecture mapping)	Nine commonly use ML (SVM, DT, kNN, MLP, AdaBoost, RF, bagging)	GBM vs. HHG (anaplastic glioma) vs. meningioma vs. PCNSL vs. BM	Adaptive boosting and random forest + advanced MRI and physiological MRI data were superior to human reading in accuracy (0.875 vs. 0.850), precision (0.862 vs. 0.798), F-score (0.774 vs. 0.740), AUROC (0.886 vs. 0.813), and classification error (5 vs. 6)	Small sample size, single MRI scanner and traditional ML
Ucuzal et al. [60]	Turkey	233	Open-source dataset from https://figshare.com (accessed on 01 January 2022).	T1C	CNN from DL algorithm, developed web-based software (Python programming language and TensorFlow, Keras, Scikit-learn, OpenCV, Pandas, NumPy, MatPlotLib, and Flask libraries)	Glioma vs. Meningioma vs. Pituitary lesions	All the calculated performance metrics are higher than 98% for classifying the types of brain tumors on the training dataset	Small size, not healthy individuals, the selection and creation of these algorithms may require a lot of time and experience
Pavabvash et al. [65]	USA	256	Institutional brain MRI database	T1, DWI, T2, FLAIR, SWI, DSC, T1C	Naïve Bayes, RF, SVM, CNN	Differentiation of posterior fossa lesions (Hemangioblastoma, Pilocytic Astrocytoma, Ependymoma, Medulloblastoma	The decision tree model achieved greater AUC for differentiation of pilocytic astrocytoma (*p* = 0.020); and ATRT (*p* = 0.001) from other types of neoplasms	Small number of rare tumor types, lack of molecular subtyping in medulloblastoma and ependymoma, manual segmentation, acquisition in different field strengths
Verma et al. [67]	Switzerland	32	Institutional brain MRI database	DSC, T1CI	DTPA-method with different texture parameters	GBM vs. PCNSL, tumefactive multiple sclerosis	The texture parameters of the original DSCE-image for mean, standard deviation and variance showed the most significant differences (*p*-value between <0.00 and 0.05) between pathologies	Small size, smaller TOI in MS, manual segmentation
Han et al. [68]	China	57	Institutional brain MRI database	T1, T2	*t*-test and statistical regression (LASSO algorithm) to develop three radiomic models base on T1 WI, T2 WI and a combination	LGG vs. multiple sclerosis	T2WI and combination models achieved better diagnostic efficacy, with AUC of 0.980, 0.988 in primary cohort and that of 0.950, 0.925 in validation cohort,	Retrospective study, small size, single scanner, unknown etiology of inflammation
Qian et al. [69]	China	412	Cancer Genome Atlas (TCGA); retrospective dataset from Beijing Tiantan Hospital	T1C	Radiomic features extraction, ML	GBM vs. single BM	SVM + LASSO classifiers had the highest prediction efficacy (AUC, 0.90)	Retrospective study; imaging data from multiple MRI systems; only CE sequences were used
Bae et al. [70]	Korea	166 (training) + 82 (validation)	retrospective institutional brain MRI database	T2, T1C	DL using radiomic features	GBM vs. single BM	DNN showed high diagnostic performance, with an AUC, sen, spec, and acc of 0.956, 90.6%, 88.0% and 89.0%	Automated tumor segmentation, not included advanced sequences, heterogeneous MR scanner types
Adu et al. [61]	China		Brain Tumor Dataset. Figshare (3064 images)	T1C	CapsNets (dilated capsulenet)	Detection + classification	Acc.: 95%	Not enough comparisons and experiments with confusion matrix
Abiwinanda et al. [43]	Indonesia		Brain Tumor Dataset. Figshare (3064 images)	T1C	CNN	Classify into three types	Acc.: 98%	Complexity of pre-processing

**Table 2:** The main characteristics and findings of the studies focused on the characterization of brain tumors.

Author and Year.	Country	N. Patients	Database	MRI Sequences and Clinical Data	AI Model	Task	Main Results	Limitations
Chang et al. [78]	USA	259	The Cancer Imaging Archives	T1, T1C, FLAIR	CNN (DL)	IDH1, 1p/19q co-deletion, MGMT	Accuracy, respectively: 94%, 92%, 83%	Small sample size; retrospective study; lack of an independent dataset
Mzoughi et al. [77]	Tunisia		BraTS 2018 dataset	T1C	3D CNN	Grade classification (LGG and HGG)	Classification accuracy: 96.49%	
Wiestler et al. [71]	Germany	37	institutional brain MRI database	T1C, FLAIR, T2, rOEF, CBV	ML (RF)	WHO grade II/III vs. WHO grade IV	Acc: 91.8%	Lack of an independent validation cohort, small sample size, retrospective study
Zhang et al. (2017) [72]	China	120	institutional brain MRI database	T1, T1C, FLAIR, ASL, DWI, DCE	ML	Comprehensive automated glioma grading scheme (LGG and HGG)	SVM is superior to the other classifiers, best performance when combined with RFE attribute selection strategy	High classification accuracy on current data but bad performance on new dataset
Kim et al. [73]	South Korea	127	retrospective institutional brain MRI database	T1, T2, FLAIR, T1C, DWI, DSC	Radiomic features extraction, ML	Glioma grading and IDH prediction	Higher performance (AUC 0.932) of multiparametric model with ADC features in tumor grading	Retrospective design, small number of patients in the validation set, data from a single institution
Cho et al. [75]	Korea	285	BraTS 2017	T1, T1C, T2, FLAIR	Radiomic features extraction, ML	Glioma grading	RF classifier showed the best performance with AUC 0.9213 for the test cohort	Not considered molecular information
Tian et al. [76]	China	153	retrospective brain MRI database	T1C, T2, DWI, ASL	Radiomic features extraction	Glioma grading (LGG vs. HGG; grade III vs. IV)	SVM model’s more promising than using the single sequence MRI for classifying LGGs from HGGs and grade III from IV	
Akkus et al. [74]	USA	159	brain tumor patient database of Mayo Clinic	T1C, T2	multi-scale CNN	1p/19q prediction	Increased enhancement, infiltrative margins, and left frontal lobe predilection are associated with 1p/19q codeletion with up to 93% accuracy	Limited original data size (solved by data augmentation)
Meng et al. [79]	China	123	Institutional brain MRI database	T1, T2, FLAIR, T1C, ADC	SVM model and 5-fold cross validation	ATRX status	AUC for ATRX mutation (ATRX(−)) on training set 0.93 (95%[CI]: 0.87–1.0), on validation set 0.84	Small dataset, lack of multiparametric MRI, just one imaging biomarker
Ren et al. [80]	China	57	Institutional brain MRI database	3D-ASL, T2, FLAIR, DWI	SVM	IDH1(+) and ATRX(−)	Accuracies/AUCs/sensitivity/specificity/PPV/NPV of predicting IDH1(+) in LGG: 94.74%/0.931/100%/85.71%/92.31%/100%; ATRX(−) in LGG with IDH1(+) 91.67%/0.926/94.74%/88.24%/90.00%/93.75%	Qualified patient population relatively small, the molecular sequencing, such as IDH2 codons, was not performed; hard to be fully understood by treating physicians and applied to routine clinical practice
Haubold et al. [82]	Germany	217	Institutional brain MRI database	T1, T1C, FLAIR	DeepMedic (CNN-based algorithm), XGBoost (SL) model for parameter optimization	ATRX, IDH1/2, MGMT, 1p19q co-deletion, LGG vs. HGG	AUC (validation/test) for LGG vs. HGG 0.981 ± 0.015/0.885 ± 0.02, ATRX(-) with AUCs of 0.979 ± 0.028/0.923 ± 0.045, followed by 0.929 ± 0.042/0.861 ± 0.023 for IDH1/2; 1p19q and MGMT achieved moderate results.	Small sample size, different MRiI manufacturer, retrospective study
Shboul et al.l [83]	USA	108	Institutional brain MRI database	T1, T1C, FLAIR, T2	XGBoost (SL model)	MGMT methylation, *IDH* mutations, 1p/19q co-deletion, *ATRX* and *TERT* mutations	The prediction models of MGMT, *IDH*, 1p/19q, *ATRX*, and *TERT* achieve a test performance AUC of 0.83 ± 0.04, 0.84 ± 0.03, 0.80 ± 0.04, 0.70 ± 0.09, and 0.82 ± 0.04, respectively	Small sample size
Calabrese et al. [84]	USA	400	Institutional brain MRI database	T1, T1C, T2, FLAIR, SWI, DWI, ASL, MD, AD, RD, and FA.	CNN, Random forest model	*mutations of IDH, TERT, TP53*, *PTEN*, *ATRX*, or *CDKN2A/B*, *MGMT* methylation, *EGFR* amplification, and combined aneuploidy of chromosome 7 and 10	Good performances; ROC AUC highest for *ATRX* (0.97) and *IDH1* (0.96) mutations	Lack of external validation

**Table 3:** Main characteristics and results of studies aimed on prognostication.

Autor and Year	Country	N. Patients	Database	MRI Sequences and Clinical Data	AI Model	Task	Main Results	Limitations
Macyszyn et al. (2019) [113]	USA	105 (retrospective) + 26 (prospective)	Hospital case series of GB at the University of Pennsylvania from 2006 to 2013	Structural, diffusion, and perfusion scans >18 years old, GBM histopathological diagnosis	Machine learning algorithm	Prediction of overall survival and molecular subtype	High prediction accuracy (survival 80%, molecular subtype 76%)	Only MRI at time of diagnosis was used in creating the predictive model, data from a single institution
Nie et al. (2019) [114]	China	68 (training dataset) + 25 (validation dataset)	Hospital case series (Huashan hospital, Shanghai, China Huashan hospital, Shanghai, China	T1 MRI, rs-fMRI and DTI HHG patients with evidence of enhancement in T1wi, no previous treatment	3D convolutional neural networks (CNNs) + support vector machine (SVM)	Prediction of overall survival	Accuracy of 90.66%	Limited clinical information (e.g., tumor genetics)
Sanghazni et al. (2022) [115]	Singapore	163 GBM patients	BraTS 2017 dataset	T1, T2, FLAIR, T1 CE	Support vector machine (SVM) classification based recursive feature elimination method to perform tumor feature selection	Prediction of overall survival	High accuracy for both 2-class and 3-class OS group predictions (89–99%)	-
Prasanna et al. (2017) [117]	USA	65 GBM patients	Cancer Imaging Archive	T1C, T2, FLAIR	402 radiomic features from enhancing lesion, PBZ and tumor necrosis	Radiomic features from the peritumoral brain zone can predict long- versus short-term survival	Features suggestive of intensity heterogeneity and textural patterns were found to be predictive of survival (*p* = 1.47 × 10^−5^) as compared to features from enhancing tumor, necrotic regions and known clinical factors	Preliminary study
Parl et al. (2020) [118]	Korea	216 patients with newly diagnosed glioblastoma: training (n = 158) and external validation sets (n = 58)	Two tertiary medical centers	DWI, perfusion	Radiomic feature selection using LASSO regression + multiparametric MR prognostic model (radiomics score + clinical predictors)	Multiparametric MR prognostic model (radiomics score + clinical predictors) vs. conventional MR radiomics model discrimination	Better discrimination (C-index, 0.74) and performance of multiparametric MRI than a conventional MR radiomics model (C-index, 0.65, *p* < 0.0001) or clinical predictors (C-index, 0.66; *p* < 0.0001); good external validation (C-index, 0.70)	Small number of patients, molecular changes were not considered in this analysis, only scans on 3.0 T
Grist et al. (2021) [119]	UK	69 participants with suspected brain tumors (medulloblastoma (N = 17), pilocytic astrocytoma (N = 22), ependymoma (considered high grade, N = 10), other tumors (N = 20)	Four clinical sites in the UK (Birmingham Children’s Hospital, Newcastle Royal Victoria Infirmary, Queen’s Medical Centre, Alder Hey Children’s Hospital, Liverpool) 2009–2017	T1, T1C, T2, DWI Many different tumor types, stages and patient ages	Unsupervised and supervised machine learning models	Perfusion, DWI, and ADC values determined two new subgroups of brain tumors with different survival characteristics (*p* < 0.01)	High accuracy (98%) by a neural network, non-invasive risk assessment tool, multi-site and multi-scanner data	Small heterogeneous cohort treated in a diverse manner, variations in scanner protocol, here are a number of children alive at study end with high-risk tumors and currently limited follow-up
Zhang et al. (2019) [130]	China	51 glioma patients who underwent radiation treatments after surgery	Hospital case series	T1, T1C, T2, FLAIR Necrosis or recurrence in different glioma subtypes, stages, location and patient ages	Deep features extracted from multimodality MRI images by two CNNs (AlexNet and Inception v3)	Distinguish glioma necrosis from recurrence in glioma patients using a radiomics model based on combinational features and multimodality MRI images	Accuracy of AlexNet and Inception v3 features is higher than that of employing handcrafted features (paired *t*-test (*p* < 0.0003)	Correlations among features were ignored, tens of thousands of features were used, the dataset used in this study was relatively small
Narang et al. (2017) [131]	USA	79 GB patients	TCGA database	Presurgical T1C, T2, FLAIR T-cell surface marker CD3D/E/G mRNA expression level data	Image-derived features extracted across the T1-post contrast and FLAIR images were selected with the Boruta package selected	Develop an imaging-derived predictive model for assessing the extent of intra-tumoral CD3 T-cell infiltration	Prediction model for CD3 infiltration achieved accuracy of 97.1% and area under the curve (AUC) of 0.993	Texture features derived only from T1-post and T2-FLAIR sequences, variation in scanning and acquisition protocols, adjustment for molecular status
Kim et al. (2018) [137]	Korea	238 patients who were pathologically confirmed as having GB and who subsequently received standard concurrent chemo-radiation therapy	Database of the local Department of Radiology between March 2011 and March 2017	T1Ci, FLAIR, DWI, and DSC imaging performed within 6 months after surgery or biopsy De novo GB diagnosis according to WHO criteria who had chemo-radiation therapy	Multiparametric radiomics selection by ANTsR and WhiteStripe packages	Distinguish progression vs. pseudoprogression	Multiparametric radiomics model (AUC, 0.90) showed better performance than any single ADC or CBV parameter, robustness (high internal and external validation)	Retrospective nature, small size of the cohort, relatively high fraction of pseudoprogression, need of validation with a 1.5T scanner, time cost and complicated analytical process
